# Introduction to computational causal inference using reproducible Stata, R, and Python code: A tutorial

**DOI:** 10.1002/sim.9234

**Published:** 2021-10-28

**Authors:** Matthew J. Smith, Mohammad A. Mansournia, Camille Maringe, Paul N. Zivich, Stephen R. Cole, Clémence Leyrat, Aurélien Belot, Bernard Rachet, Miguel A. Luque–Fernandez

**Affiliations:** 1 Inequalities in Cancer Outcomes Network, Department of Non-communicable Disease Epidemiology, London School of Hygiene and Tropical Medicine, London, UK; 2 Department of Epidemiology and Biostatistics, Tehran University of Medical Sciences, Tehran, Iran; 3 Department of Epidemiology, University of North Carolina at Chapel Hill, Chapel Hill, North Carolina, USA; 4 Carolina Population Center, University of North Carolina at Chapel Hill, Chapel Hill, North Carolina, USA; 5 Non-communicable Disease and Cancer Epidemiology Group, Instituto de Investigacion Biosanitaria de Granada (ibs.GRANADA), Andalusian School of Public Health, University of Granada, Granada, Spain; 6 Biomedical Network Research Centers of Epidemiology and Public Health (CIBERESP), Madrid, Spain

**Keywords:** causal inference, double-robust methods, g-formula, G-methods, inverse probability weighting, machine learning, propensity score, regression adjustment, targeted maximum likelihood estimation

## Abstract

The main purpose of many medical studies is to estimate the effects of a treatment or exposure on an outcome. However, it is not always possible to randomize the study participants to a particular treatment, therefore observational study designs may be used. There are major challenges with observational studies; one of which is confounding. Controlling for confounding is commonly performed by direct adjustment of measured confounders; although, sometimes this approach is suboptimal due to modeling assumptions and misspecification. Recent advances in the field of causal inference have dealt with confounding by building on classical standardization methods. However, these recent advances have progressed quickly with a relative paucity of computational-oriented applied tutorials contributing to some confusion in the use of these methods among applied researchers. In this tutorial, we show the computational implementation of different causal inference estimators from a historical perspective where new estimators were developed to overcome the limitations of the previous estimators (ie, nonparametric and parametric g-formula, inverse probability weighting, double-robust, and data-adaptive estimators). We illustrate the implementation of different methods using an empirical example from the Connors study based on intensive care medicine, and most importantly, we provide reproducible and commented code in Stata, R, and Python for researchers to adapt in their own observational study. The code can be accessed at https://github.com/migariane/Tutorial_Computational_Causal_Inference_Estimators.

## 1 | INTRODUCTION

Often, questions that motivate studies in the health, social, and behavioral sciences are causal. However, these research questions are usually answered using classical statistical methods, including multivariable outcome regression, to assess the relationship between an exposure and an outcome. For example, in a given population, what is the mortality risk difference among those patients who received surgery for colorectal cancer vs those who did not?^1^ Often, the associations between a treatment and an outcome assessed using classical methods cannot be interpreted as causal. Randomized clinical trials (RCTs) are considered the gold standard for causal inference because randomization ensures the outcome is independent of the treatment assignment. RCTs are not always feasible (ie, for ethical reasons or when the interest lies in the estimation of real-world effects) or may fail when randomization does not work. Therefore, when causality cannot be guaranteed by design (ie, in observational studies) or when the randomization procedure fails, causal inference methods must be used. Based on the randomized experiment setting, Rubin introduced the potential outcomes framework: extending causal inference from randomized experiments to observational data.^2^ Then, these methods were extended to observational settings with time-varying confounders.^3^

One of the aims when designing an observational study is to answer a scientific question that characterizes the effect of a treatment on an outcome. This question is translated to an *estimand* (a target), which is the as yet unknown quantity we are interested in. Then, we use the *estimator* (a method), which is an algorithm that uses the values of the observations in the sample (in other words, a function of the random variables) to generate the *estimate* (the quantitative value generated for the estimator). The estimators are represented by algebraic equations that explicitly describe a function of the realized observations. Over the years, rapid ongoing advances in the field of causal inference have resulted in several algorithms that improve upon classical methods (ie, outcome regression adjustment) to estimate the causal effect of a treatment on an outcome. These methods incorporate estimators using propensity scores, g-computation, or a combination of both (ie, double-robust estimators). G-computation methods model the outcome mechanism, whereas propensity-score based methods model the treatment allocation, thus balancing the treatment groups in terms of the confounders. Often, double-robust estimators are preferred over classical single-robust regression approaches when the research question is causal.^4,5^

In this tutorial, we introduce the estimators mentioned above and show their computational implementation in regards to their chronological development (ie, the methods were developed to address the limitations of the previous approaches). However, these methods are also introduced from a practical computational perspective, allowing readers to learn by using the replicable code. We use the Stata statistical software (StataCorp, 2020. StataCorp LLC, College Station, TX), R statistical software (R Development Core Team, 2020. *R: A language and environment for statistical computing*. R Foundation for Statistical Computing, Vienna, Austria), and Python software (Python Software Foundation, 2020). All materials are available at a GitHub repository for reuse and replication of our examples at https://github.com/migariane/TutorialComputationalCausalInferenceEstimators. All examples in this article use Stata code, but examples using R and Python are provided on the GitHub repository.

In the following sections, we will illustrate the computational implementation of different estimators that are computing the same estimand (ie, the average treatment effect (ATE), a.k.a risk difference for a binary treatment and outcome). We will not focus on the assessment of heterogeneous treatment effects. In Section 2, we briefly introduce the setting to estimate the ATE using Connors’ study. In Section 3, we introduce the g-computation based on the g-formula; and in Section 4, we introduce the methods based on the inverse probability of treatment weights (IPTW). Afterwards, in Section 5, we describe the computation of double-robust methods including the augmented inverse probability of treatment weighting (AIPTW), and in Section 6, we present targeted maximum likelihood estimation (TMLE). Finally, in Section 7, we compare the performance of the various estimators using a single simulated data set.

## 2 | SETTING TO ESTIMATE THE ATE

To illustrate the implementation of the most common causal inference estimators we use an empirical data set from the prospective cohort study of Connors et al.^6^ We use the data within the aforementioned GitHub repository; the original data are available at: https://hbiostat.org/data/, however some variables will need to be recoded (see Box 1). The study was set within intensive care units (ICUs) of five US teaching hospitals between 1989 and 1994, and evaluated the effectiveness of right heart catheterization (RHC) on short-term mortality (30 days) of 5735 critically ill adult patients (2184 treated and 3551 untreated) receiving care for 1 of 9 prespecified disease categories.

A common estimand in causal inference is the ATE. The ATE is defined by an average of the difference of two random variables (ie, the potential outcomes Y(1) and Y(0)).^3,7,8^ For a binary treatment, each patient in the study has two potential outcomes (ie, Y(a)), where Y(1) denotes the potential outcome if they received RHC, and Y(0) denotes the potential outcome if they did not receive RHC (Supplementary Appendix 1).^2,3,7,8^ More detailed introductions of the causal language used for the potential outcomes, and the assumptions needed to estimate causal effects using observational data, we refer readers to a recently published tutorial.^9^ In our illustration, the outcome is short-term mortality (a binary variable) defined as mortality within 30 days after ICU admission; the main intervention was RHC. We define the vector (W) to include the set of predefined confounders. To estimate the ATE (ie, the standardized short-term risk difference of death for those patients who received RHC versus those who did not), we compute different estimators using the prospective cohort study of Connors et al.^6^

**FIGURE 1:**
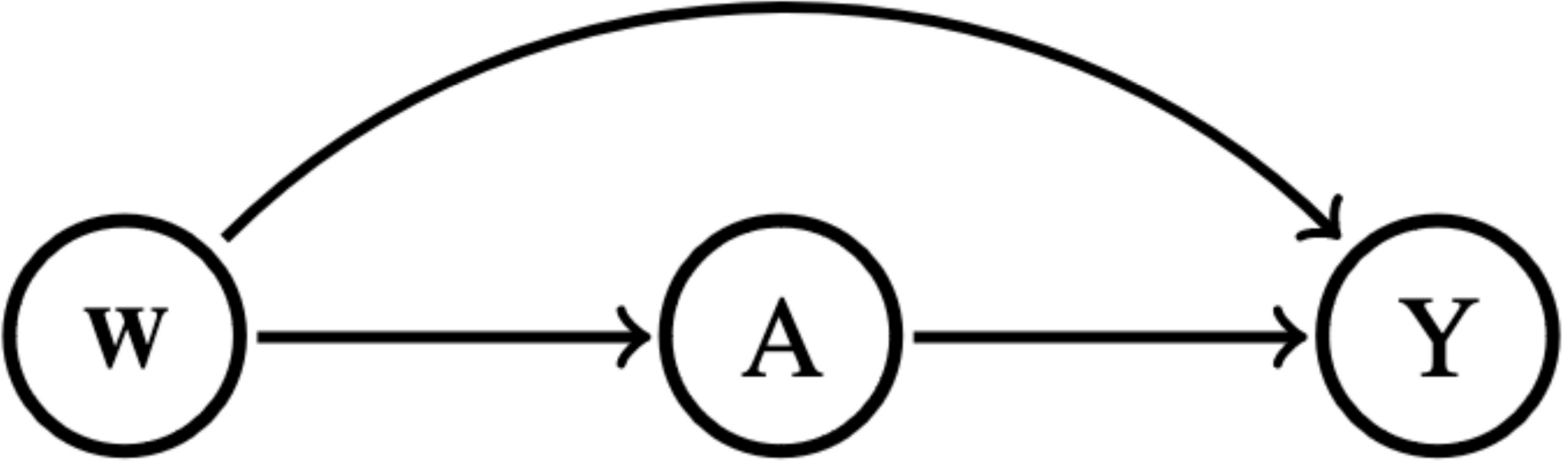
Y: outcome; A: treatment; W: sufficient set of variables to control for confounding, as outlined in Connors et al^6^

Figure 1 is a directed acyclic graph representing the causal relationship between the vector of predefined confounders (ie, W: sex, age, education, race, and cancer), the intervention (A: receipt of RHC during their stay at the ICU), and the outcome (Y: vital status of the patient in an ICU at 30 days after admission). Note that throughout the article, we refer to A as the “treatment,” but it can be used interchangeably with the terms “exposure” or “intervention” depending on the context.

To estimate the ATE of the intervention (RHC) on short-term mortality, we assume counterfactual consistency, conditional exchangeability, non-interference, and positivity (see Supplementary Appendix 1). Furthermore, all the variables included in W are confounders of the effect of A on Y; there are no intermediate variables (ie, mediators or colliders); and there is no residual confounding. Therefore, we assume, for illustrative purposes, that the set of covariates included in W suffices, implying that the assumption of conditional mean independence holds (ie, sufficient control for confounding).

## 3 | G-COMPUTATION METHODS BASED ON THE G-FORMULA

### 3.1 | Nonparametric g-formula

Regression adjustment is used to estimate the main effect of a risk factor on an outcome. It is one of the classical methods used in epidemiology to control for confounding. When a regression model does not include interactions the use of regression adjustment to control for confounding makes the assumption that the effect is constant across levels of confounders (W) included in the model.^5^ Note that we focus on a binary outcome and treatment, thus “classical methods” will involve logistic regression adjustment to estimate the conditional odds ratio (OR) for the association between the treatment and the outcome. However, the OR is a non-collapsible measure of association, which means that the conditional OR cannot be used to estimate the marginal ATE.^5^ Furthermore, in observational and randomized studies, the estimate of the effect measure can be confounded given the different distribution of individual characteristics by treatment levels; thus, causal inference methods are needed to correct for the imbalance. For example, in the instance of differential age distributions between two treatment groups, classical methods will approach the problem using multivariable regression adjustment. However, causal inference methods use the g-formula, a generalization of the classical standardization procedure, which allows obtaining an unconfounded marginal estimation of the ATE. For a binary treatment, the g-formula is given by:^3^

(1) $$\text{ATE}=\underset{w}{\sum }[P(Y=1|A=1,W=w)-P(Y=1|A=0,W=w)]P(W=w),$$

where

$$P(Y=1|A=a,W=w)=\frac{P(W=w,A=a,Y=1)}{\sum_{y}P(W=w,A=a,Y=y)}$$

is the conditional probability of the outcome Y=1, given the treatment A=a, and the set of confounders W=w. Note, the implementation of the g-formula requires the use of the total law of probability.^2^ In probability theory, the law of total probability is a fundamental rule relating marginal probabilities to conditional probabilities.

In the following set of boxes, we show how to estimate the marginal causal effect (ie, effect of RHC on short-term mortality) using the nonparametric and parametric g-formula in Stata. The Stata code, and the implementation of the same computational approach using R and Python, is provided in a GitHub repository: https://github.com/migariane/Tutorial_Computational_Causal_Inference_Estimators. For now, in the first nine boxes, we use sex as the sole confounder, namely “c” (sex: 0 female, 1 male). It is an oversimplification for pedagogical purposes, which allows readers to readily appreciate the implementation of the computation of the parametric and nonparametric g-formula using G-computation methods. In Boxes 10 to 13, we extend the methods by including multiple confounders. In contrast to classical methods (regression-based methods), the way we adjust for confounding based on the generalization of standardization (g-formula) is more coherent as we assume that the effect of RHC on short-term mortality can differ by sex. Classical methods, by including an interaction term in the model, can allow the effect to differ by sex but this hampers the interpretation of the main effect of RHC.^5^ It is a subtle difference but provides a richer adjustment for confounding.

In Box 1, we declare the global variables Y, A, C, and W to match the presented algebraic nomenclature (ie, Y: outcome, A: treatment, C: one unique confounder, and W: a set of confounders). We use these global variables throughout the implementation of the different methods.

Box 1: **Setting the data**


1 clear
2 set more off
3 use “rhc.dta” // (*From GitHub repo:* https://github.con/migariane/
*TutorialComputationalCausallnferenceEstimators*)
4 * Define the outcome (Y), exposure (A), confounder (C), and confounders (W)
5 lab def rhc 0 “No RHC” 1 “RHC”, modify // *Define labels for the rhc variable*
6 lab val rhc rhc // *Assign the label to the rhc variable*
7 global Y death_d30 // *Outcome: 30-day mortality*
8 global A rhc // *Treatment: Right Heart Catheterisation*
9 global C sex // *One unique confounder of the set of W*
10 global W i.sex c.age c.edu i.race i.carcinoma // *A set of five confounders*

We first introduce, in Box 2, a naïve approach to estimate the ATE: we regress the outcome over the treatment (using a linear model) and adjust for the confounder (ie, sex). In the naïve regression adjustment, the interpretation of the value for the regression coefficient of the treatment in the model is assumed to be constant for a fixed level of the confounder (ie, sex). In Box 2, the result for the naive regression adjustment shows strong evidence (*p* < 0.001) that the risk difference of death within 30 days is 7.35% higher among those with RHC (95% confidence interval [CI]: 4.84–9.86), conditional on sex. Results for this method, and for all of the methods in this tutorial, are shown in Table 1.

Box 2: **Adjusted regression**


1 regress $Y $A $C // *Risk difference = 7.35%; 95% CI: (4.84 – 9.86); p<0.001*
2 *// Bootstrap 95% CI*
3 qui bootstrap, reps(1000) seed(1): regr $Y $A $C

For the first causal inference method we use, in Box 3, we compute the marginal probability of the confounder, save it, and generate two new variables named *sexf* for females and *sexm* for males (ie, the marginal proportion of females was 44%, thus 56% are males, which shows unequal probability of being assigned the treatment by sex). We then compute, and save in a matrix, the expected conditional probabilities of the outcome by levels of the treatment and the confounder. We substitute the results of the matrix into the g-formula, given in Equation (1), and compute the ATE.

Box 3: **Nonparametric g-formula for the ATE**


1 proportion $C			// *Marginal probability of C (sex)*
2 matrix m=e(b)
3 gen sexf = m[1,1]
4 sum sexf
5 gen sexm = m[1,2]
6 sum sexm
7 ssc install sumup
8 sunup $Y, by($A $C)		// *Expected conditional probabilities of the outcome by levels of A and C*
9	// *from sumup command extract the conditional means by the given levels of A and C (i.e. zero and one*)
10 matrix y00 = r(stat1)	// *[6,1] matrix for E(Y/A=0, C=0)*
11 matrix y01 = r(stat2)	// *[6,1] matrix for E(Y/A=0, C=1)*
12 matrix y10 = r(Stat3)	// *[6,1] matrix for E(Y/A=1, C=0)*
13 matrix y11 = r(Stat4)	// *[6,1] matrix for E(Y/A=1, C=1)*
14 // *see “matrix list y00”: position subscript [3,1] is the one of interest*
15 *// Applying the g-formula*
16 gen EY1 = ((y11[3,1]-y01(3,1]))*sexm // *E(Y/A=1)*
17 gen EYO = ((y10[3,1]-y00[3,1]))*sexf // *E(Y/A=0)*
18 qui: mean EY1 EYO
19 matrix ATE = r(table)
20 display “The ATE is: “ ATE[1,1] + ATE[1,2] // *Applying the g-formula*
21 drop EY1 EYO
22 // *Also one can try*
23 gen ATE = ((y11[3,1]-y01[3,1]))*sexm + ((y10[3,1]-y00[3,1]))*sexf
24 qui sum ATE
25 drop ATE
26
27 // *Check that Stata “teffects” command obtains the same estimate*
28 teffects ra ($Y $C) ($A)
29 // *The ATE from “teffects” implementation is: 7.37 (95% CI 4.83 – 9.91)*

For the case of only one confounder, the results from the naïve regression adjustment and g-formula approaches are the same to one decimal place and nearly the same for the multivariable setting (ie, multiple confounders) (Table 1). However, this is due to the use of a teaching data set with limited residual confounding (ie, good balance of treatment across the levels of the confounders). Note that in real settings it will not be the case and more importantly the results and interpretation will differ (ie, conditional vs marginal estimate). The naïve approach (Box 2) is a conditional estimation interpreted as the individual risk for treated vs non-treated, holding the levels of the confounder constant. Whereas, the g-formula is a marginal contrast (Box 3) and therefore it must be interpreted at a population level.

The interpretation of the estimate from the naïve approach (Box 2) is difficult to conceptualize because we are holding the value of the confounder constant, and it requires the assumption that the effect of the treatment is the same for males and females (ie, constant across the levels of the confounders).^5,10^ However, in observational studies, the ATE within strata of confounders may differ. Therefore, the g-formula has become a powerful alternative to the multivariable regression adjustment when controlling for confounding and evaluating the effects of treatments.^3^

#### 3.1.1 | Statistical inference: The bootstrap

When constructing confidence intervals from an estimate obtained from a causal inference estimator, model-based standard errors (SE) are incorrect. This is because the model-based SE do not account for the different steps we need to take when we balance the confounders between treatment groups. We use the bootstrap procedure for inference implemented in Stata with the command *bootstrap*.^11^ The bootstrap is a resampling method used to approximate the variance of the estimate (eg, G-computation for the ATE).^11,12^ When estimating the variance using the bootstrap method, the observed data is thought of as representing the entire target population, and each draw (with replacement) from the data mimics the sampling variability. Under certain assumptions, this set of draws will return estimates of the sampling distribution that are equivalent to having actually repeated the sampling from the original target population.^11^ Typically, for procedures that use parametric models, the bootstrap is a reliable estimator of the variance (ie, the bootstrap uses the standard deviation of the bootstrap estimates of the ATE as a plug-in for the SE and the computation of the confidence intervals). However, note, it does not account for the bias engendered by model misspecification, so it only provides sampling variability for whatever the estimator is estimating.^11^ The accuracy with which the bootstrap distribution estimates the sampling distribution depends on the number of observations in the original sample and the number of replications in the bootstrap.

To implement the bootstrap procedure in Stata we need to define a program that estimates the nonparametric g-formula and then samples (with replacement) the ATE to derive the confidence intervals for the ATE. In Box 4 we provide the code to compute the SE for the ATE using Stata.

Box 4: **Bootstrap 95% confidence intervals (CI) for the ATE estimated using the nonparametric g-formula**


1 capture drop program ATE
2 program define ATE, rclass	// *As before but now define a program to estimate the ATE*
3	capture drop ATE
4	sumup $Y, by($A $C)
5	matrix y00 = r(Stat1)
6	matrix y01 = r(Stat2)
7	matrix y10 = r(stat3)
8	matrix y11 = r(Stat4)
9	gen ATE = ((y11[3,1]-y01[3,1]))#sexm + ((y10[3,1]-y00[3,1]))*sexf
10	qui sum ATE
11	return scalar ate = `r(mean)’
12 end
13 qui bootstrap r(ate), reps(1000) seed(1): ATE // *Bootstrap 1000 estimates of the ATE*
14 estat boot, all
15 drop ATE

Based on the nonparametric g-formula, the estimate of the ATE was 7.37%. Using the command “estat boot, all,” Stata gives three sets of CIs for the ATE; by default the bootstrap procedure will only provide the Normal-based CI. The first (N) is an approximation based on the Normal distribution (95% CI: 4.79–9.94). The naïve approach also uses the Normal approximation based on the central limit theorem giving asymptotic CIs. It is observed that the performances of the bootstrap CIs are better than the asymptotic confidence intervals in terms of the nominal coverage. Furthermore, the average length of bootstrap CIs is slightly larger than those of asymptotic CIs.^13,14^ The second (P) is based on the percentile of the bootstrap distribution (95% CI: 4.59–9.82), and the third (BC) is based on the bias-corrected (95% CI: 4.72–9.89) (Table 1). Note that the percentile interval is a simple “first-order” interval that is formed from quantiles of the bootstrap distribution. However, it is based only on bootstrap samples and does not adjust for skewness in the bootstrap distribution, unlike the bias-corrected. Thus, we will report the BC 95% CI.^14^

For an alternative implementation of the nonparametric g-formula, we turn our attention to computing the ATE using a fully saturated regression model (still with only one confounder) using the full information of the sample (including the interactions between the treatment and the confounders). In Stata, there are two different approaches that we illustrate in Boxes 5 and 6 using the commands *predictnl* and *margins*.

To estimate the ATE using a fully saturated regression model we need to include all the possible interactions between the treatment and the different levels of the confounders (if categorical, otherwise with a continuous confounder) (Box 5). We do this by using the hashtag “#” symbol in Stata to include the interaction between A and C. The Stata prefix “*ibn.*” specifies estimation of a categorical variable without the use of a base level (use with the *noconstant* option). The prefix “c.()” indicates that the confounder (ie, sex) is to be used as a continuous variable (it does not matter for continuous or binary variables, but will matter for categorical variables). The *coeflegend* option asks Stata to provide the list of the labels of the variables in the analysis. The labels are then used for the *predictnl* command, which allows the computation of the nonparametric predictions based on the combination of the conditional probabilities from the regression coefficients. Finally, we average over the predictions to get the nonparametric estimate for the ATE. Note that the approach introduced in Box 5, in contrast to the approach presented in Box 3, is less computationally intensive in terms of time and code.

Box 5: **Nonparametric g-formula using a fully saturated regression model in Stata (A)**


1 regress $Y ibn.$A ibn.$A#$C, noconstant vce(robust) cosflegend
2 predictnl ATE = (_b[1.rhc] + _b[1.rhc#1.sex]*sex) - (_b[0bn.rhc] + _b[0bn.rhc#1.sex]*sex)
3 qui sum ATE
4 display “The ATE is: “ `r(mean)’
5 drop ATE
6 
7 // *Bootstrap 95% CI*
8 capture program drop ATE
9 program define ATE, rclass
10 capture drop ATE
11	regress $Y ibn.$A ibn.$A#$C, noconstant vce(robust) coeflegend
12   predictnl ATE = (_b[1.rhc] + _b[1.rhc#1.sex]*sex) - (_b[0bn.rhc] + _b[0bn.rhc#1.sex)*sex)
13   qui sum ATE
14   return scalar ate = `r(mean)’
15 end
16 qui bootstrap r(ate), reps(1000) seed(1): ATE
17 estat boot, all
18 drop ATE

A simpler option for the nonparametric g-formula would be to use the *margins* command to estimate the marginal probabilities using the option *vce*(*unconditional*) (Box 6). Then, the difference in marginal probabilities between the treated vs non-treated is implemented using the contrast option from the *margins* command. Note that here we obtain the same estimate of the ATE as 7.37% (95%: CI 4.83–9.91) but the appropriate 95% CI has been calculated using the delta method (Table 1). The delta method is a statistical approach to derive the SE of an asymptotically normally distributed estimator. It uses a first-order Taylor approximation, which is how we approximate the distribution of a function using a tangent line (ie, the first derivative).^15^ Therefore, using the delta method here we assume that the ATE estimate from the G-computation is normally distributed.^16^

Box 6: **Nonparametric g-formula using a fully saturated regression model in Stata (B)**


1 regress $Y ibn.$A ibn.$A#$C, noconstant vce(robust) // *Fully saturated model specification*
2 margins $A, vce(unconditional) // *Marginal probability for A*
3 margins r.$A, contrast(nowald) // *Difference in marginal probability between treatment groups*

### 3.2 | Parametric g-formula

In contrast to the nonparametric methods (ie, probability distribution free or infinite dimensions), parametric methods are not affected by the curse of dimensionality.^17^ However, to compute the ATE parametrically we have to assume there is a particular probability distribution that fits the distribution of our data. To compute the ATE, we first regress (using a simple linear regression model) the outcome over the confounder(s) separately for each treatment group. We then predict the probability of treatment and contrast the difference in the expected probabilities between the two treatment groups (note that every individual has two predicted probabilities corresponding to the two estimated potential outcomes). The algebraic form of the ATE under the G-computation is given by

(2) $$\text{ATE}=\frac{1}{n}\sum_{i=1}^{n}\left(E\left(Y_{i}|A_{i}=1,W_{i}\right)-E\left(Y_{i}|A_{i}=0,W_{i}\right)\right).$$

In Box 7, we provide the code to compute, by hand, the ATE based on the parametric g-formula for one confounder using parametric regression adjustment (based on formula 2).

Box 7: **Parametric regression adjustment implementation of the g-formula**


1 regress $Y $C if $A==1	// *Expected probability amongst those with RHC*
2 predict double y1hat
3 regress $Y $C if $A==0	// *Expected probability amongst those without RHC*
4 predict double y0hat
5 mean y1hat y0hat		// *Difference between the expected probabilities*
6 lincom _b[y1hat] - _b[y0hat]  // *ATE and biased confidence interval*

The risk of mortality among those with RHC is 7.37%, higher compared to those without RHC. Note that using a simple linear combination (ie, lincom command in Stata) to compute a 95% CI for the linear contrast between the marginal potential outcomes results in a biased CI that does not account for the two-step procedure to get the marginal probabilities.

In Box 8, we confirm the result we obtained (by hand in Box 7) using the STATA’s *teffects* command and including the “*ra*” option to perform the regression adjustment. Note the difference between the naïve and the *teffects* 95% CIs. The *teffects* uses the delta method to correct for the uncertainty for each of the two models (ie, E(Y∣A=1,C) and E(Y∣A=0,C)), and provide appropriate statistical inference.

Box 8: **Parametric regression adjustment using Stata’s *teffects***


1 teffects ra ($Y $C) ($A) // *parametric g-formula implementation in Stata*

With the *teffects* command in Stata the ATE is 7.37%, which is the same as we obtained by hand (Box 7). However, note that the 95% CI for the ATE using the command from Stata (*teffects*) is more conservative than using the naïve approach without accounting for the uncertainty of the two regression models to predict the marginal probabilities (ie, 95% CI: 4.83–9.91 and 95% CI: 7.35–7.39, respectively for the *teffects* and the naïve approaches) (Table 1).

Again, if we want to compute the 95% CI by hand using Stata we could use the bootstrap procedure (refer to Box 4 for an explanation).

Box 9: **Bootstrap for the parametric regression adjustment**


1 capture program drop ATE
2 program define ATE, rclass	// *Define the program that will run the bootstrap*
3	capture drop y1		// *Drop any previously defined variable ‘y1’*
4	capture drop y0		// Drop any previously defined variable *‘y0’*
5	reg $Y $C if $A==1	// *Regress the outcome amongst those with A=1*
6	predict double y1, xb	// *Generate a variable (y1) to hold the predicted values*
7	quiet sum y1		// *Summarise the predicted value of the regression model*
8	reg $Y $C if $A==0	// *Regress the outcome amongst those with A=0*
9	predict double y0, xb	// *Generate a variable (y0) to hold the predicted values*
10	quiet sum y0		// *Summarise the predicted value of the regression model*
11	mean y1 y0		// *Check the mean for y1 and for y0*
12	lincom _b[y1]-_b[y0]	// *Calculate the difference in mean between y1 and y0*
13	return scalar ate = `r(estimate)’ // *Save the value of the difference in mean in a scalar called ‘ate’*
14 end
15 qui bootstrap r(ate), reps(1000) seed(1): ATE // *Bootstrap 1000 times to generate the standard errors*
16 estat bootstrap, all		// *Reports a table summarising the results of the bootstrap*
17 drop ATE

After bootstrapping, the estimate of the ATE is 7.37% and the bias-corrected 95% CI: (4.68–9.84) (Table 1).

As is often the case, there is almost always more than one confounder. The parametric computation of the g-formula can easily be extended to include more than one confounder: remember that W includes a set of five confounders.

Box 10: **Parametric multivariable regression adjustment implementation of the g-formula**


1 regress $Y $W if $A==1	// *Regression model with all confounders for those with RHC*
2 predict double y1hat 
3 regress $Y $W if $A==0	// *Regression model with all confounders for those without RHC*
4 predict double y0hat 
5 mean y1hat y0hat		// *ATE is the difference in expectations*
6 lincom _b[y1that] - _b[y0hat])// *We use lincom for the contrast*
7				// *but it gives a biased confidence interval for the ATE*

The ATE of those with RHC (ie, risk of mortality among those with RHC) is 8.36%, (95% CI: 8.25–8.47) higher compared to those without RHC in contrast to the naïve regression multivariable adjustment of 8.26% (Table 1). Note, the 95% CI provided by the *lincom* Stata command is biased as it is not accounting for the two-step estimation procedure to derive the ATE.

In Box 11, we use Stata’s *teffects* command to confirm our results. We now include W instead of the single confounder C.

Box 11: **Parametric multivariable regression adjustment using Stata’s *teffects* command**


1 teffects ra ($Y $W) ($A)

We obtain the same results with Stata’s *teffects* command as with our calculations by hand (ATE 8.36%; 95% CI: 5.83–10.88). However, note again the difference between the 95% CI estimated naïvely and using the *teffects* command (Table 1).

In Box 12, we show another way of obtaining the ATE under the parametric g-formula approach using the Stata *margins* command after fitting a fully saturated regression model. First, we regress the dependent variable (Y) over the treatment (A), including the interaction of *A* with all of the other confounders (W). We do this using the same approach as in Box 5 (ie, ibn.$A#c.($W)) to include the interaction between all levels of A and a vector of all of the other confounders included in the model. Then, the *margins* command calculates the predicted value of the expectation of the outcome given the treatment and the confounders, and reports the mean value of those predictions for each level of the treatment (A) (ie, E(Y∣A=1,W) and E(Y∣A=0,W)). Finally, to compute the ATE and provide corrected 95% CI based on the delta method, we use the *contrast* option to compute the ATE. The ATE is the difference in the average 30-day mortality between those treated with RHC and those who were not (ie, E(Y∣A=1,W)−E(Y∣A=0,W)). Note the results are the same as before using the *teffects* command (ie, ATE 8.36%; 95% CI: 5.83–10.88) (Table 1).

Box 12: **Parametric multivariable regression adjustment using Stata’s *margins*command**


1 regress $Y ibn.$A ibn.$A#($W), noconstant vce(robust) // *Fully saturated model*
2 margins $A, vce(unconditional) // *E(Y/A=1,W), E(Y/A=0,W) and Delta method for the standard errors (i.e., vce unconditional) and 95%CI*
3 margins r.$A, contrast(nowald) // *ATE and Delta method for the standard error and 95%CI*

Finally, in Box 13, we show how to compute the bootstrap 95% CIs for the G-computation implementation of the g-formula by hand using regression adjustment in Stata.

Box 13: **Bootstrap for the multivariable parametric regression adjustment**


1 capture program drop ATE
2 program define ATE, rclass
3	capture drop y1
4	capture drop y0
5	reg $Y $W if $A==1
6	predict double y1, xb
7	quiet sum y1
8	reg $Y $W if $A==0
9	predict double y0, xb
10	quiet sum y0 
11	mean y1 y0 
12	lincom _b[y1])-_b[y0]
13	return scalar ate = `r(estimate)’
14 end
15 qui bootstrap r(ate), reps(1000) seed(1): ATE dots
16 estat boot, all
17 drop ATE

After bootstrapping, the estimate of the ATE is 8.36%. The bootstrapped bias-corrected 95% CI is (5.68–10.84) (Table 1).

## 4 | INVERSE PROBABILITY OF TREATMENT WEIGHTING

### 4.1 | Inverse probability weighting based on the propensity score

In observational studies, some individuals will be more likely than others to be treated (*A* = 1) due to their characteristics. Suppose some individuals who were treated were unlikely to be treated based on a specific set of features encapsulated in a particular vector of confounders (W). To balance the differences in characteristics between treatment groups, we reweight the outcome variable of these individuals by the inverse of their probability of the treatment (*A*) actually received (ie, propensity score). Originally, the weights were motivated from the classical Horvitz and Thompson survey estimator used to reweight the outcome variable by the inverse probability that it is observed, thus accounting for the sampling process.^18^ The result of this weighting procedure is that, among the treated we up-weight those who had a low probability of being treated, and among the untreated we up-weight those who were unlikely to be untreated; that is, the individuals underrepresented in their treatment group. As a consequence, the weighted set of data is unchanged apart from A and W are now conditionally independent. Therefore, a comparison of $Y_{w}(1)$ to $Y_{w}(0)$ gives a marginal causal effect under the three identification assumptions (Supplementary Appendix 1) while also assuming the propensity score model is correctly specified. The inverse probability of treatment weighting (IPTW), and the g-formula when targeting the same estimand (ie, the ATE), are equivalent in the nonparametric setting.^3,19^ In Supplementary Appendix 2, we provide a proof of the equivalence between IPTW and G-computation procedures using the law of total expectation.

Departing from the identification assumptions of the ATE for the regression adjustment G-computation estimand $(ATE=E_{w}(E(Y|A=1,W)-E_{w}(Y|A=0,W)$), we can rewrite the same estimand as a function of the distribution of A given W (ie, P(A=1∣W) a.k.a propensity score or treatment mechanism).

Therefore, the estimator is given by

(3) $$ATE=\frac{1}{n}\sum_{i=1}^{n}\left(\frac{A_{i}}{P\left(A_{i}=1|W_{i}\right)}-\frac{1-A_{i}}{\left(1-P\left(A_{i}=1|W_{i}\right)\right)}\right)Y_{i}.$$

There is a modified version (ie, Hájek type)^20^ of the IPTW estimator (Equation 3) consisting of stabilized weights, which is more commonly used in practice when treatment and exposure vary over time (ie, time dependent confounding). However, stabilized weights should have a mean of 1, but some values could be higher (ie, large weights). The stabilized version of the IPTW estimator is given by

(4) $$\text{ATE}=\frac{\sum \left(\frac{AY}{P(A=1|W)}\right)}{\sum \left(\frac{A}{P(A=1|W)}\right)}-\frac{\sum \left(\frac{(1-A)Y}{1-P(A=1|W)}\right)}{\sum \left(\frac{\left(1-A\right)}{1-P(A=1|W)}\right)}.$$

In Box 14, we show how to compute the IPTW by hand in two steps:

- First, the propensity score model is fitted in rows 1 to 4 (ie, a logistic regression model for a binary treatment)
- Then the sampling weights are generated based on the inverse probability of treatment actually received. Note, the weights are just the implementation of the classical Horvitz-Thompson survey estimator,^18^ (see rows 3 and 4) also known as unstabilized weights (rows 5–9).

When there are near violations of the positivity assumption, the unstabilized weights can have large values, forcing the variance to increase and exacerbate the uncertainty of the ATE estimation. Therefore, it is advisable to explore the distribution of the weights to evaluate the extent to which they balance the distribution of confounders across the levels of the treatment (ie, equally distributed). It is common to provide a table with the unweighted and weighted differences of the standardized means of the confounders by the levels of the treatment. Also, it is common to visualize an overlap of the propensity scores by the level of the treatment to identify and visualize positivity or near positivity violations and to explore the descriptive distribution of the weights (ie, mean, minimum and maximum values). Lastly, while we are showing the use of logistic regression, the propensity score model may alternatively be estimated using nonparametric approaches (eg, the *twang*^21^ R package uses generalized boosted regression modeling).

Box 14: **Computation of the IPTW estimator for the ATE**


1 logit $A $W, vce(robust) nolog	// *Propensity score model for the treatment*
2 predict double ps			// *Propensity score prediction*
3 generate double ipw1 = ($A==1)/ps	// *Sampling weights for the treated group*
4 generate double ipw0 = ($A==0)/(1-ps) // *Sampling weights for the non-treated group*
5 mean $Y (pw=ipw1], coeflegend		// *Weighted outcome probability among treated*
6 scalar Y1 = _b[(death_d30]
7 mean $Y [pw=ipw0], coeflegend		// *Weighted outcome probability among non treated*
8 scalar Y0 = _b[death_d30]
9 display “ATE =“ Y1 - Y0

The risk difference between those with RHC and those without is 8.33%. Re-weighting the individuals generates a pseudo-population (weighted population) from which the data generation does not follow a theoretical distribution and individuals are no longer independent. Therefore the 95% CI is estimated using the bootstrap procedure in Box 15.

As before, we can obtain confidence intervals using the bootstrap procedure.

Box 15: **Bootstrap computation for the IPTW estimator**


1 capture program drop ATE
2 program define ATE, rclass
3	capture drop y1
4	capture drop y0
5	capture drop ipw0
6	capture drop ipw1
7	capture drop ps
8	logit $A $W, vce(robust) nolog	// *propensity score model for the exposure*
9	predict double ps // *propensity score predictions*
10	generate double ipw1 = ($A==1)/ps // *Sampling weights for the treated group*
11	generate double ipw0 = ($A==0)/(1-ps) // *Sampling weights for the non-treated group*
12	regress $Y [pw=ipw1] // *Weighted outcome probability among treated*
13	matrix y1 = e(b)
14	gen double y1 = y1[1,1]
15	regress $Y [pw=ipw0] // *Weighted outcome probability among non-treated*
16	matrix y0 = e(b)
17	gen double y0 = y0[1,1]
18	mean y1 y0
19	lincom _b[y1]-_b[y0]
20	return scalar ate = ‘r(estimate)’
21 end
22 qui bootstrap r(ate), reps(1000) seed(1): ATE
23 estat boot, all
24 drop ATE

After bootstrapping, the estimate of the ATE is 8.33%. The bootstrapped bias-corrected confidence interval is: (5.65–10.81) (Table 1).

We now confirm this result in Box 16 using Stata’s *teffects* command. Note that the Horvitz-Thompson estimator is implemented using the *ipw* option. We obtain the same point estimate for the ATE and slightly different, but consistent, 95% CI based on the robust SE derived from the functional delta method (ie, ATE 8.33%; 95% CI: 5.81–10.85) (Table 1).

Box 16: **Computation of the IPTW estimator for the ATE using Stata’s *teffects* command**


1 teffects ipw ($Y) ($A $W, logit), nolog vsquish

In Box 17, we show how to explore the balance of the confounders after weighting the contributions of individuals using IPTW (ie, that the distribution of the confounders are balanced between those with RHC and those without). When applying weights, we must be careful as we are assuming that the treatment has been balanced across the levels of the confounders. In Stata, we use the *tebalance* option after using the *teffects* command but the balance can be assessed by hand as well.

Box 17: **Assessing IPTW balance**


1 qui teffects ipw ($Y) ($A $W)
2 tebalance summarize // *Stata’s tebalance*
3 
4 // *tebalance by hand (sex)*
5 egen sexst = std(sex) // *Standardisation*
6 logistic $A $W // *Propensity score*
7 predict double ps
8 gen ipw = .
9 replace ipw=($A==1)/ps if $A==1
10 replace ipw=($A==0)/(1-ps) if $A==0
11 regress sexst $A // *Raw difference*
12 regress sexst $A ([pw=ipw] // *standardised difference*

After weighting, the two treatment groups appear to be well-balanced. Prior to weighting, there was some imbalance (absolute values of the standardized differences close to, or beyond, 0.10) on sex, education level and presence/extent of cancer between treatment groups.^22^ A variance ratio (ie, the ratio of the standardized distribution of the confounders by the levels of the treatment) equal to 1 before and after weighting informs us that the distribution of the confounders across the levels of the treatments is the same (ie, perfectly balanced). Note, the weighted variance ratio for the continuous variable age is 0.79, which is slightly further from 1 than the variance ratio for the original (unweighted) sample (ie, 0.82); this slight change is possibly because the weighted mean for age might have greater sampling variance than the unweighted mean (Table 2).^23^

There is no definitive value at which the treatment is considered unbalanced; however, as a guideline, a variance ratio less than 0.5 indicates that the data is not balanced and the potential for the positivity violation must be explored (ie, when P(A=a∣C=c) is near to zero or one). An additional strategy is to check the distribution of the weights: if there are very large weights this indicates the violation of the positivity assumption but, also, it can be due to parametric modeling misspecification. Again there is no clear consensus but, when there are very large weights, researchers often set the weights to a less extreme value. This is done by trimming or removing the data at the extremes of the distribution of the weights (eg, the 5th and 95th percentiles).^24^ Trimming the weights reduces variance (ie, omitting the largest weights and making the positivity assumption more plausible), but at the expense of introducing bias.^25^ However, another alternative without dropping observations is truncation, whereby all the values of the weights, larger than a user-specified maximum value or percentile (eg, 1st and 99th or 5th and 95th), are replaced by that threshold value.^25,26^ In extreme cases, when the weights are extremely large, changing the estimand could be another solution (eg, estimating the ATE in a subset of the sample, just like among only those treated, representing the average treatment effect among the treated -ATT-).

**FIGURE 2:**
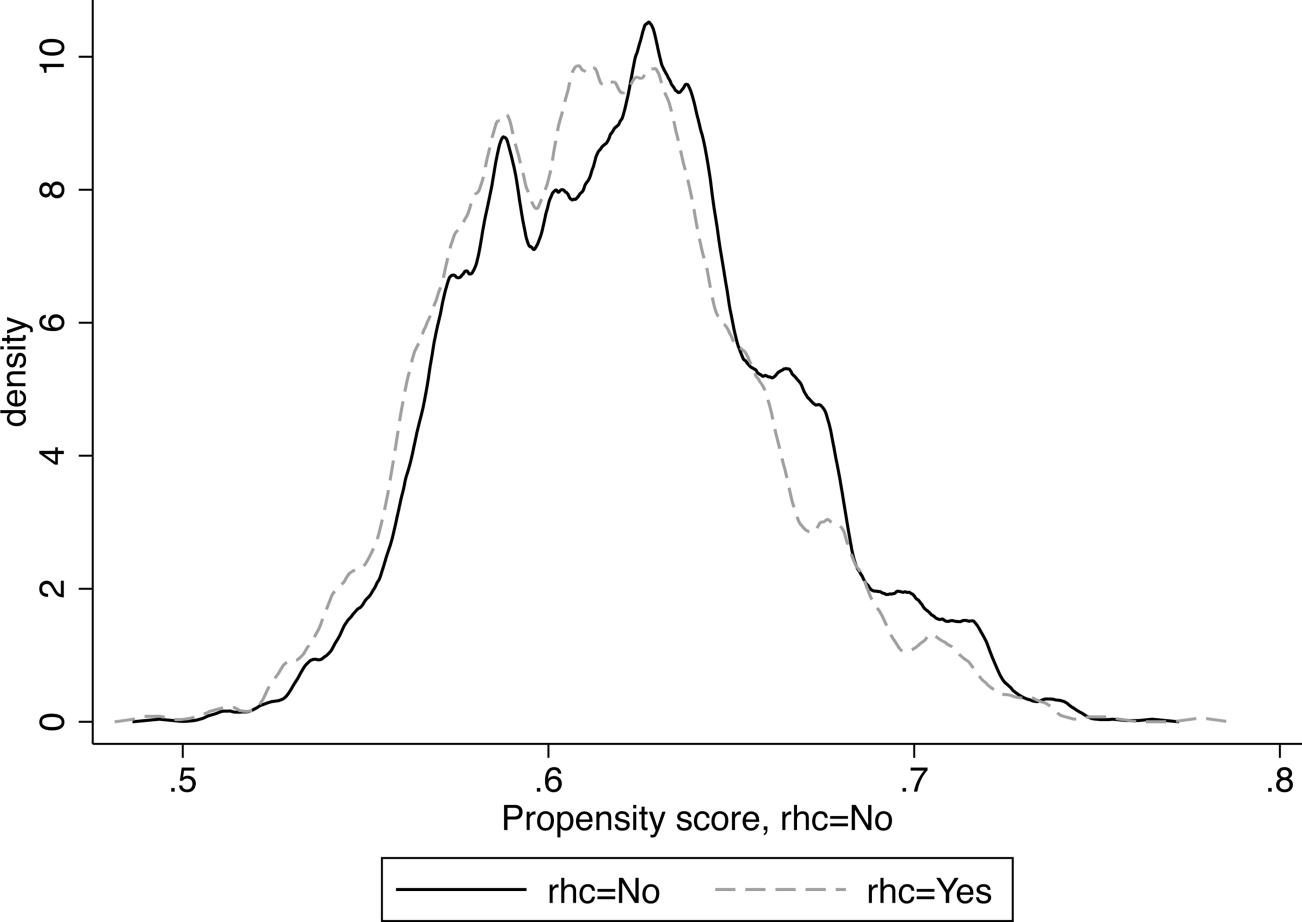
Propensity score overlap by treatment status

It is also important to check the overlap of the propensity scores of the two treatment groups. The “overlap” gives a visual identification regarding the strength of confounding and whether it is acceptable. In Box 18, we show how to visualize the “overlap” using a kernel density estimate of the treatment assignment by the levels of the treatment. Figure 2 shows there is a suitable amount of overlap.

Box 18: **Assessing IPTW overlap by hand**


1 sort $A
2 by $A: summarize ps
3 kdensity ps if $A==1, generate(x1pointsa d1A) nograph n(10000) // *Nonparametric kernel density estimate of the distribution of the propensity score among treated individuals*
4 kdensity ps if $A==0, generate(x0pointsa d0A) nograph n(10000) // *Nonparametric kernel density estimate of the distribution of the propensity score among non-treated individuals*
5 label variable d1A “density for RHC=1”
6 label variable d0A “density for RHC=0”
7 twoway (line d0A x0pointsa , yaxis(1))(line d1A x1pointsa, yaxis(2))

The overlap plots can be obtained with Stata’s *overlap* command after calling the *teffects* command. We are using data where there is a good balance and overlap but in real-world observational data the balance and overlap, before using any weighting procedure, are not likely to be well balanced.

Box 19: **Assessing overlap using teffects overlap**


1 qui: teffects ipw ($Y) ($A $W, logit), nolog vsquish
2 teffects overlap

### 4.2 | Marginal structural model with stabilized weights

We now introduce the marginal structural model (MSM) as a transition to the double-robust methods.^27^ An MSM is a marginal mean model. A popular method for estimating the parameters of the MSM is weighted regression modeling that estimates the marginal distributions of the counterfactuals.^27,28^ In the MSM, the coefficient for the treatment is the estimate of treatment effect, usually the ATE. The MSM uses an updated version of the Horvitz-Thompson weights, commonly used in sampling theory.^18^ The weights represent the inverse of the probability of treatment (a.k.a propensity score). In Box 20, we show how to compute a MSM:

- First, in rows 1 to 18, we compute the propensity score and the weights.
- In row 20, we fit the MSM using the unstabilized weight, and in row 21, using the stabilized version. The approach to compute the weights is equivalent to the one presented in Box 14 where reweighting the individuals generated a pseudo-population and classical statistical inference does not hold.^29^ Thus, for statistical inference we use the *vce*(*robust*) option, which implements the delta method, to estimate the appropriate SE for the ATE.^17^ However, using the bootstrap procedure is also a valid option.
- Finally, in rows 21 to 45, we show how to implement the bootstrap procedure to compute the 95% CI.

The ATE derived from the MSM is 8.33%, and the 95% CI using the delta method: (5.77–10.89) and (5.84–10.85) using the bootstrap procedure (Table 1).

Box 20: **Computation of the IPTW estimator for the ATE using a MSM**


1 // *baseline treatment probabilities*
2 logit $A, vce(robust) nolog
3 predict double nps, pr
4 // *propensity score model*
5 logit $A $W, vce(robust) nolog
6 predict double dps, pr
7 // *Unstabilised weight*
8 gen ipw = .
9 replace ipw=($A==1)/dps if $A==1
10 replace ipw=($A==0)/(1-dps) if $A==0
11 sum ipw
12
13 // *Stabilised weight*
14 gen sws = .
15 replace sws = nps/dps if $A==1
16 replace sws = (1-nps)/(1-dps) if $A==0
17 sum sws
18
19 // *MSM*
20 reg $Y $A [pw=ipw], vce(robust) // *MSM unstabilised weight*
21 reg $Y $A [pw=sws], vce(robust) // *MSM stabilised weight*
22
23 // *Bootstrap the 95% confidence intervals*
24 capture program drop ATE
25 program define ATE, rclass
26 capture drop nps
27 capture drop dps
28 capture drop sws
29    // *Baseline treatment probabilities*
30	logit $A, vce(robust) nolog
31	predict double nps, pr
32	// *propensity score model*
33	logit $A $W, vce(robust) nolog
34	predict double dps, pr
35	// *Stabilized weight*
36	gen sws = .
37	replace sws = nps/dps if $A==1
38	replace sws = (1-nps)/(1-dps) if $A==0
39	sum sws
40	// *MSM*
41	reg $Y $A [pw=sws], vce (robust)
42	return scalar ate = e(b)[1,1]
43 end
44 qui bootstrap r(ate), reps(1000) seed(1): ATE
45 estat boot, all

## 5 | DOUBLE-ROBUST METHODS

### 5.1 | Inverse probability weighting plus regression adjustment

The IPTW-RA is an estimator using a G-computation regression adjustment (RA) that incorporates the estimated stabilized IPTW. It has been shown that the IPTW-RA helps to correct the estimator when the regression function is misspecified, provided that the propensity score model for the treatment is correctly specified. When the regression function is correctly specified, the weights do not affect the consistency of the estimator even if the model from which they are derived is misspecified.^30^ Note that combining both, the IPTW and the RA approaches, the IPTW-RA estimator has the special property that it is consistent as long as at least one of the two models (ie, ITPW and RA) is correctly specified, it is why estimators that combine both modeling approaches are named double-robust.^31^ When one uses G-computation methods only, they rely on extrapolation of the treatment effects when there are identifiability issues due to data sparsity and near-positivity violations. Adding the IPTW to the regression adjustment allows evaluation of the balance of the treatment and of possible positivity violations, increasing the researcher’s awareness of the limitations of causal inference modeling. It is encouraged, when possible, to explore the implementation of the nonparametric g-formula (using the important confounders) and identify potential problems with the data relating to the curse of dimensionality from finite samples (ie, zero empty cells for a given combination of conditional probabilities from the different variables included in analysis needed to implement the g-formula).

Although IPTW with regression adjustment (IPTW-RA) is usually more efficient than IPTW, it also relies on different parametric modeling assumptions: (i) a parametric G-computation regression adjustment model, and (ii) a model for the propensity score of binary treatments. The G-computation weighted model uses the weights calculated from the predictions of the propensity score logistic model. An estimated propensity score that is close to 0 or 1 is problematic, since it implies that some individuals will receive a very large weight leading to imprecise and unstable estimates (ie, near positivity assumption violation). Therefore, the use of stabilized weights is suggested (see code from Box 20), and the bootstrap for statistical inference.

Box 21: **Computation of the IPTW-RA estimator for the ATE and bootstrap for statistical inference**


1 capture program drop ATE
2  program define ATE, rclass
3	capture drop y1
4	capture drop y0
5	// *Weighted (stabilised weights) regression adjustment among the treated*
6	reg $Y $W if $A==1 (pw=sws)
7	predict double y1 , xb
8	quiet sum y1 
9	return scalar y1 =`r(mean)’
10	// *Weighted (stabilised weights) regression adjustment among the non-treated*
11	reg $Y $W if $A==0 [pw=sws]
12	predict double y0, xb
13	quiet sum y0
14	return scalar y0=`r(mean)’
15	mean y1 y0
16	// *ATE*
17	lincom _b[y1]-_b[y0]
18	return scalar ate = `r(estimate)’
19 end
20 qui bootstrap r(ate), reps(1000) seed(1): ATE // *Bootstraping for statistical inference*
21 estat boot, all

After bootstrapping, the estimate of the IPTW-RA ATE is 8.35%, bias-corrected 95% CI (5.83 – 10.87). The results are very similar to those obtained using the Stata’s *teffects* command with the option *ipwra* presented in Box 22 (ie, ATE: 8.35% and 95% CI: 5.82–10.87) (Table 1).

Note that using *ipwra* we specify two models (ie, the model for the outcome and the model for the treatment).

Box 22: **Computation of the IPTW-RA estimator for the ATE using Stata’s *teffects***


1 teffects ipwra ($Y $W) ($A $W), nolog vsquish

### 5.2 | Augmented inverse probability of treatment weighting

The AIPTW estimator is an improved IPTW estimator that includes an augmentation term, which corrects the estimator when the treatment model is misspecified. When the treatment model is correctly specified, the augmentation term vanishes as the sample size becomes large. Thus, the AIPTW estimator is more efficient than the IPTW. However, like the IPTW, the AIPTW does not perform well when the predicted treatment probabilities are too close to zero or one (ie, near positivity violations). Under correct modeling specification, the augmentation term has expectation zero and includes the expectation of the propensity score and the regression adjustment outcome. Thus, the AIPTW combines two parametric models (ie, a model for the outcome and a model for the treatment).^32,33^ The AIPTW estimator produces a consistent estimate of the ATE if either of the two models has been correctly specified.^30,33^

Focusing on the IPTW estimator for the ATE in Equation (3), let ${\hat{\mu }}_{a}$ be the expectation of the ATE using IPTW, more formally this is

$${\hat{\mu }}_{a}=E\left(\frac{I(A=a)}{g(A|W)}Y\right),$$

where I is the indicator function and g(.) refers to the treatment mechanism.

We can rewrite the equation in the form of an estimating equation (see Glossary) as,

$$\frac{1}{n}\sum_{i=1}^{n}\left(\frac{I\left(A_{i}=a\right)Y_{i}}{g\left(A_{i}|W_{i}\right)}-\mu_{a}\right)=0,$$

As long as the estimating function has mean zero then $\hat{\mu }$ is a consistent estimator of μ, where $\mu_{a}=E(Y|A=a,W)$. If we augment the estimating function using a mean-zero term,

$$\frac{I(A=a)-g(A=a|W)}{g(A=a|W)},$$

including the propensity score expectation (g(A=a∣W)), we have integrated both the estimation of the treatment mechanism and the mean outcome (E(Y∣A=a,W)), then

$$E\left(\frac{I(A=a)Y}{g(A=a|W)}-\left(\frac{I(A=a)-g(A=a|W)}{g(A=a|W)}\right)E(Y|A=a,W)\right)-\mu_{a}=0.$$

Rearranging the equation we can see that the AIPTW estimator is a combination of inverse weighting and outcome regression defined for a binary treatment as

(5) $$\frac{1}{n}\underset{\text{G-computation-Regression-Adjustment}}{\underset{︸}{\sum_{i=1}^{n}\left(E\left(Y_{i}|A_{i}=1,W_{i}\right)-E\left(Y_{i}|A_{i}=0,W_{i}\right)\right)}}+\frac{1}{n}\sum_{i=1}^{n}\underset{\text{Zero-expectation}}{\underset{︸}{\left(\frac{A_{i}\left[Y_{i}-E\left(Y_{i}|A_{i}=1,W_{i}\right)\right]}{g\left(A_{i}=1|W_{i}\right)}-\frac{\left(1-A_{i}\right)\left[Y_{i}-E\left(Y_{i}|A_{i}=1,W_{i}\right)\right]}{g\left(A_{i}=0|W_{i}\right)}\right)}},$$

where the ATE from the AIPTW estimator is defined as

(6) $$\text{AIPTW-ATE}=\mu_{1}-\mu_{0},\;\;\mu_{1}=\frac{1}{n}\sum_{i=1}^{n}\left(E\left(Y_{i}|A_{i}=1,W_{i}\right)+\frac{A_{i}\left[Y_{i}-E\left(Y_{i}|A_{i}=1,W_{i}\right)\right]}{g\left(A_{i}=1|W_{i}\right)}\right),\;\;\mu_{0}=\frac{1}{n}\sum_{i=1}^{n}\left(E\left(Y_{i}|A_{i}=0,W_{i}\right)+\frac{\left(1-A_{i}\right)\left[Y_{i}-E\left(Y_{i}|A_{i}=1,W_{i}\right)\right]}{g\left(A_{i}=0|W_{i}\right)}\right).$$

The second term in Equation (5) can be interpreted as playing the role of two nuisance parameters of the AIPTW estimating function. The nuisance parameters are represented as a weighted sum of the residuals for the conditional mean of the outcome.^16^ Equation (5) shows that the AIPTW estimator equals the g-formula estimator if the outcome model is correctly specified irrespective of the treatment model. Likewise, the point estimate will be equal to the IPTW estimator if the treatment model is correctly specified, irrespective of the outcome model.^31,33^

In Box 23 we show how to compute the AIPTW estimator for the ATE using Stata:

- Step 1: First, we predict the mean outcome by treatment status using G-computation regression adjustment (rows 1–7).
- Step 2: Then, we compute the inverse of treatment weights (rows 9–16).
- Step 3: Using Equation (5), we compute the ATE (rows 18–26).
- Step 4: Finally, we compute 95% CI using the bootstrap procedure in Stata (rows 28–52).

Box 23: **Computation of the AIPTW estimator for the ATE and bootstrap for statistical inference**


1 // *Step (i) prediction model for the outcome using G-computation regression adjustment*
2 qui glm $Y $A $W, fam(bin)
3 predict double QAW, mu
4 qui glm $Y $W if $A==1, fam(bin)
5 predict double Q1W, mu
6 qui glm $Y $W if $A==0, fam(bin)
7 predict double Q0W, mu
8
9 // *Step (ii): prediction model for the treatment*
10 qui logit $A $W
11 predict double dps, pr
12 qui logit $A
13 predict double nps, pr
14 gen sews = .
15 replace sws = nps/dps if $A==1
16 replace sws = (1-nps)/(1-dps) if $A==0
17 
18 // *Step (iii): Estimation equation based on analytical formula 5*
19 gen double y1 = (sws*($Y-QAW) + (Q1W))
20 quiet sum y1
21 gen double y0 = (sws*($Y-QAW) + (Q0W))
22 quiet sum y0
23 mean y1 y0
24 lincom _b[y1] - _b[y0]
25 
26 // *step (iv) Bootstrap confidence intervals*
27 capture program drop ATE
28 program define ATE, rclass
29 capture drop y1
30 capture drop y0
31 capture drop Q*
32 qui glm $Y $A $W, fam(bin)
33 predict double QAW, mu
34 qui glm $Y $W if $A==1, fam(bin)
35 predict double Q1W, mu
36 qui glm $Y $W if $A==0, fam(bin)
37 predict double Q0W, mu
38 gen double y1 = (sws*($Y-QAW) + (Q1W))
39 quiet sum y1
40 return scalar y1=`r(mean)’
41 gen double y0 = (sws*($Y-QAW) + (Q0W))
42 quiet sum y0
43 return scalar y0=`r(mean)’
44 mean y1 y0
45 lincom _b[y1] - _b[y0]
46 return scalar ate =`r(estimate)’
47 end
48 qui bootstrap r(ate), reps(1000) seed(1): ATE
49 estat boot, all
50 drop ATE

After bootstrapping the ATE is 8.39% and the bias-corrected 95% CI confidence intervals are: (5.87–10.89) (Table 1). In Box 24, we show the same results using Stata’s *teffects* command with the *aipw* option. Note that we have to specify the model for the treatment and the model for the outcome.

Box 24: **Computation of the AIPTW estimator for the ATE using Stata’s *teffects***


1 teffects aipw ($Y $W) ($A $W, logit), nolog vsquish

The ATE is 8.35%, 95% CI: 5.82 to 10.87 (Table 1).

## 6 | DATA-ADAPTIVE ESTIMATION: ENSEMBLE LEARNING TARGETED MAXIMUM LIKELIHOOD ESTIMATION

Targeted maximum likelihood estimation (TMLE) is a plug-in, semi-parametric, double-robust method that reduces the bias of an initial estimate by allowing for flexible estimation using nonparametric data-adaptive machine-learning methods to target an estimate closer to the true model specification.^4^ There are several TMLE tutorials published elsewhere,^34–38^ but here we provide a brief introduction. To learn more about the algorithm, readers can refer to van der Laan and Rose’s TMLE book,^4^ and from a practical perspective to a step-by-step tutorial illustrated in a realistic cancer epidemiology scenario published by *Statistics in Medicine* in 2018.^38^ The advantages of TMLE have been demonstrated in both simulation studies and applied analyses.^4,39^ Evidence shows that TMLE can provide the least biased ATE estimate compared with other double-robust estimators such as the IPTW-RA and AIPTW. In particular, while TMLE and AIPW estimators are asymptotically equal, TMLE enjoys better finite sample properties. Separately, TMLE is often implemented with ensemble machine learning, which can relax model specification constraints.^4,39^

In Box 25, we provide the computational implementation of TMLE by hand (without data-adaptive estimation) to guide and interpret the different steps involved in the TMLE. A description of the theory behind these steps can be found elsewhere.^38^

- Step 1: We estimate the expected outcome given treatment and confounders (E(Y∣A,W): this is called the plug-in initial estimate of the estimator obtained via G-computation, namely $Q_{0}$ (Box 25: rows 1–15).
- Step 2: We define the expected treatment given the confounders as we did previously for the estimation of the propensity score in Box 14, namely $g_{0}$. Steps 1 and 2 are similar to the double-robust methods of AIPTW; however, we now come to the advantage of TMLE (Box 25: rows 17–21).
- Step 3: We regress the predicted treatment values and predicted outcome introduced in the model as an offset on the observed outcome. The parameter estimates (epsilon) for that regression are used to correct the initial estimations of $Q_{0}$ (Box 25: rows 24–27). In other words, we reduce the residual bias and optimized the bias-variance trade-off for the estimate of the ATE so that we can obtain valid statistical inference. Note that the TMLE framework adds the possibility to estimate the $Q_{0}$ and $g_{0}$ models using data adaptive machine learning algorithms and selecting the best model or an ensemble of the models.^4^ It has been shown that using machine learning algorithms reduces misspecification bias.^40^ Note, in Box 25, the residual bias is reduced by solving an equation that calculates how much to update, or fluctuate, our initial outcome estimates
$$E_{*}[Y|A,W]=logit(E[Y|A,W])+\epsilon H(A,W),$$
  where $E_{*}[Y|A,W]$ represents the updated initial expectation of the outcome (Y) given the treatment status (A) and the set of confounders (W). To solve this equation, we fit an intercept-free logistic regression (using *H* as the only predictor of the observed outcome) and the initially predicted outcome (under the observed treatment) as an offset (Step 3: rows 24–27) as a targeting step aimed to reduce bias. Fitting the logistic regression, using maximum likelihood procedures, TMLE yields many useful statistical properties, such as: (1) the final estimate is consistent as long as either the outcome or treatment model are estimated correctly (consistently); (2) if both of these models are estimated consistently, the final estimate achieves “semi-parametric efficiency,” that is, variance reduction as the sample size approaches infinity. Also the AIPTW is semi-parametric efficient.
- Step 4: We added the coefficient 𝜖 of the clever covariate H in the previous step to the expected outcome for all observations from the model fitted in Step 1 using (Step 4: rows 29–31), updating the $Q_{0}$ model predictions to $Q_{1}$.
$$\begin{array}{c} Q_{1}(A=1,W)=E_{*}[Y|A=1,W])=expit(logit(E[Y|A=1,W])+\epsilon H(1,W)),\;\text{and}\;\\ Q_{1}(A=0,W)=E_{*}[Y|A=0,W])=expit(logit(E[Y|A=0,W])+\epsilon H(0,W)). \end{array}$$
- Step 5: We compute the ATE as the difference between expectations of the updated $Q_{1}$ predictions in the previous step (ie, E[Y∣A=1,W])−E[Y∣A=0,W])) (Box 25: rows 33–36). It is worth noting that Steps 3 and 4, which are improvements to AIPTW and IPTW-RA estimators, are the very concepts that make TMLE more robust against near positivity violations and force the estimator to respect the boundaries of the limits of the parameter space (ie., the probabilities stay between 0 and 1). For example, to estimate the ATE using the AIPTW estimator the researcher sets the estimation equation equal to zero. However, solving the estimating equation when there are near violations of the positivity assumption can cause the estimator to fall outside the boundaries of the parameter space (ie, 0 and 1). Using TMLE, the ATE estimate is 8.34%, 95% CI: 5.82–10.98 (Table 1), which is consistent with all the previous estimates using different estimators.
- Finally, in Step 6, we provide statistical inference using the functional delta method and the influence function (IF).^4,16,41,42^ In the next section we briefly introduce these concepts.

Box 25: **Computational implementation of TMLE by hand**


1 * *Step 1: prediction model for the outcome Q0 (G-computation)*
2	glm $Y $A $W, fam(binomial)
3	predict double QAW_0, mu
4	gen aa=$A
5	replace $A = 0
6	predict double Q0W_0, mu
7	replace $A = 1
8	predict double Q1W_0, mu
9	replace $A = aa
10	drop aa
11
12	// *Q to logit scale*
13	gen logQAW = log(QAW / (1 - QAW))
14	gen logQ1W = log(Q1W / (1 - Q1W))
15	gen logQ0W = log(Q0W / (1 - Q0W))
16
17 * *Step 2: prediction model for the treatment g0 (IPTW)*
18	glm $A $W, fam(binomial)
19	predict gw, mu
20	gen double H1W = $A / gw
21	gen double H0W = (1 - $A) / (1 - gw)
22
23 * *Step 3: Computing the clever covariate H(A,W) and estimating the parameter (epsilon) (MLE)*
24	glm $Y H1W H0W, fam(binomial) offset (logQAW) noconstant
25	mat a = e(b)
26	gen eps1 = a[1,1]
27	gen eps2 = a[1,2]
28
29 * *Step 4: update from Q0W and Q1W to Q0W_1 and Q1W_1*
30	gen double Q1W_1 = exp(eps1 / gv + logQ1W) / (1 + exp(eps1 / gv + logQ1W))
31	gen double Q0W_1 = exp(eps2 / (1 - gw) + logQ0W ) / (1 + exp(eps2 / (1 - gw) + logQ0W))
32
33 * *Step 5: Targeted estimate of the ATE*
34	gen ATE = (Q1W_1 - Q0W_1)
35	summ ATE
36	global ATE = `r(mean)’
37
38 * *Step 6: Statistical inference (functional Delta method): Influence function*
39	qui sum(Q1W_1)
40	gen EY1tmle = `r(mean)’
41	qui sum(Q0W_1)
42	gen EY0tmle = `r(mean)’
43 
44	gen d1 = (($A * ($Y - Q1W_1)/gw)) + Q1W_1 - EY1tmle
45	gen d0 = ((1 - $A) * ($Y - Q0W_1)/(1 - gw)) + Q0W_1 - EY0tmle
46 
47	gen IF = d1 - d0
48	qui sum IF
49	gen varIF = r(Var) / r(N)
50 
51	global LCI = $ATE - 1.96*sqrt(varIF)
52	global UCI = $ATE + 1.96*sqrt(varIF)
53	display “ATE:” %05.4f $ATE _col(15) “95%CI: “ %05.4f $LCI “,” %05.4f $UCI

### 6.1 | Statistical inference for data-adaptive estimators: Functional delta method

We used the bootstrap procedure and delta method for statistical inference presetting the previous estimators. Although both approaches are commonly used in practice, and show good statistical properties in a wide range of settings, they have some limitations. The bootstrap procedure is computationally intensive for large data sets and the use of the delta method will not always be appropriate (ie, nonparametric settings). Furthermore, when data-adaptive estimation is used, the bootstrap procedure is not supported theoretically, and the functional delta method based on the IF is required. The IF is a fundamental object of semi-parametric theory that allows us to characterize a wide range of estimators and their efficiency.^4,16,42^ The IF of a regular asymptotic and linear estimator $\hat{\psi }$ of ψ(θ), where θ is a random variable based on independent and identically distributed samples $O_{i}$ which capture the first order asymptotic behavior of $\hat{\psi }$, such that

$$n^{\frac{1}{2}}\hat{\psi }-\psi (\theta )=n^{-\frac{1}{2}}\sum_{i=1}^{n}\text{IF}\left(O_{i};\theta \right)+o_{p}(1),$$

where $O_{p}(1)$ represents the remainder term from the first order approximation that converges to zero (in terms of the probability) when the sample size converges to infinity. Mathematically, we can identify the IF as being the second term of a first degree Taylor approximation.^41,43^ From the variance of the IF we derive the SE of the ATE from the TMLE estimator. Therefore, the functional delta method based on the IF readily allows the application of the central limit theorem and, therefore, to compute Wald-type confidence intervals.^4^ However, using the IF for statistical inference may require larger sample sizes to avoid finite-sample issues. Recent research and theoretical developments support the use of double-robust cross-fit estimators to retain valid statistical inference when using machine learning algorithms that are non-Donsker.^44^ The computation of the IF is provided in Box 25 (Step 6: rows 38–53).

In Box 26, we outline how to compute the ATE using data-adaptive procedures implemented in the *eltmle* user-written Stata command.^45^ This command implements the TMLE framework for the ATE of the marginal risk ratio and odds ratio for a binary or continuous outcome and a binary treatment. It also includes the use of data-adaptive estimation of the propensity score *g*_0_ and regression outcome $Q_{0}$ models via ensemble learning,^46^ which is implemented by calling the *SuperLearner* package v.2.0–21 from R.^46,47^ The super-learner uses 5-fold cross-validation by default to assess the performance of prediction regarding the potential outcomes and the propensity score as weighted averages of a set of machine learning algorithms. The *SuperLearner* has default algorithms implemented in the base installation of the tmle-R package v.1.2.0–5.^35^ The default algorithms include the following: (i) stepwise selection, (ii) generalized linear modeling (GLM), (iii) a GLM variant that includes second order polynomials and two-by-two interactions of the main terms included in the model. Additionally, *eltmle* has an option to include Bayes generalized linear models and generalized additive models as additional algorithms.

Box 26: **TMLE and data-adaptive estimation with Stata’s user written *eltmle***


1	ssc install eltmle //*install via ssc or “github install migariane/eltmle” via GitHub*
2	help eltmle // *Description of the command*
3	clear
4	set more off
5	use “rhc.dta”, clear
6	global W sex age edu race carcinoma
7	eltmle $Y $A $W, tmle bal // *check balance*

The ATE is 8.35%, 95% CI: 5.82–10.87 (Table 1).

## 7 | SIMULATION

The motivation of this section is to compare all of the different methods provided in the tutorial under a simple Monte Carlo simulated experiment. For simplicity and pedagogical purposes, we only simulate one sample. However, we provide the results and code in R of a Monte Carlo experiment with 1000 samples based on the same template as the one presented here and available at https://github.com/migariane/TutorialComputationalCausalInferenceEstimators. In Box 27, we outline the data generation process to create random variables including the confounders, the treatment, and the outcome. Afterward, we estimate the simulated value for the ATE, and compute the ATE using all the aforementioned different estimators under a scenario of forced near-positivity violation and model misspecification. Lastly, we compare their performance based on the relative bias with respect to the value of the simulated ATE (note that this approximates bias, as we only simulate 1 data set). Note that other metrics to assess performance can also be used, including the variance of the estimate. The simulation setting includes a binary outcome (Y), potential outcomes (ie, Y(1) and Y(0)), and a binary treatment (A). The vector of confounders W reflect the commonly analyzed cancer patient characteristics: deprivation level (w1, five categories), age at diagnosis (w2, binary), cancer stage (w3, four categories), and comorbidity (w4, four categories).

Box 27: **Data generation for the Monte Carlo experiment**


1 // *Data generation*
2   clear
3   set obs 1000
4   set seed 777
5   gen w1 = round(runiform(1, 5)) //*Quintiles of Socioeconomic Deprivation*
6   gen w2 = rbinomial(1, 0.45) //*Binary: probability age >65 = 0.45*
7   gen w3 = round(runiform(0, 1) + 0.75*(w2) + 0.8*(w1)) //*Stage*
8   recode w3 (5/6=1) //*Stage (TNM): categorical 4 levels*
9   gen w4 = round(runiform(0, 1) + 1.2*(w2) + 0.2*(w1)) //*Comorbidites: categorical four levels*
10  gen A = (rbinomial(1,invlogit(−3 – 0.5*(w4) + 1.5*(w2) + 0.75*(w3) + 0.25*(w1) + 0.8*(w2)*(w4)))) // *Binary treatment*
11  gen Y1 = (invlogit(−3 + 1 + 0.25*(w4) + 0.75*(w3) + 0.8*(w2)*(w4) + 0.05*(w1))) // *Potential outcome 1*
12  gen Y0 = (invlogit(−3 + 0 + 0.25*(w4) + 0.75*(w3) + 0.8*(w2)*(w4) + 0.05*(w1))) // *Potential outcome 2*
13  gen psi = Y1-Y0 // *Simulated ATE*
14  gen Y = A*(Y1) + (1 - A)*Y0 // *Binary outcome (consistency)*
15 
16	// *Estimate the true simulated ATE*
17  mean psi
18 
19 // *ATE estimation*
20  * Regression adjustment
21  teffects ra (Y i.w1 i.w2 i.w3 i.w4) (A)
22  estimates store ra
23 
24  * IPTW
25  teffects ipw (Y) (A i.w1 i.w2 i.w3 i.w4)
26  estimates store ipw
27 
28  * IPTW-RA
29  teffects ipwra (Y i.w1 i.w2 i.w3 i.w4) (A i.w1 i.w2 i.w3 i.w4)
30  estimates store ipwra
31 
32  * AIPTW
33  teffects aipw (Y i.w1 i.w2 i.w3 i.w4) (A i.w1 i.w2 i.w3 i.w4)
34  estimates store aipw
35 
36  * Results
37  qui reg psi
38  estimates store psi
39  estout psi ra ipw ipwra aipw
40 
41  // *Ensemble learning maximum likelihood estimation*
42  eltmle Y A w1 w2 w3 w4, tmle bal
43 
44  // *Relative bias for the ATE*
45 
46  * Regression adjustment
47    display abs(0.1652804 – 0.1726079 )/0.1652804
48    0.04433375 // *4.4% bias*
49  * IPTW
50    display abs(0.1652804 – 0.1597895)/0.1652804
51    0.03322173 // *3.3% bias*
52  * IPTW-RA
53    display abs(0.1652804 – 0.1673554)/0.1652804
54    0.01255442 // *1.2% bias*
55  * AIPTW
56    display abs(0.1652804 – 0.1682798)/0.1652804
57    0.01814734 // *1.8% bias*
58  * ELTMLE
59    display abs(0.1652804 – 0.1652167)/0.1652804
60    0.00038541 // *0% bias to 3 decimal places*

For a single-instance simulated data set, compared to the true ATE of 0.165, all of the methods produced a biased estimate under near positivity violations and model misspecification (ie, RA: 4.4% bias, IPTW: 3.3% bias, IPTW-RA: 1.2% bias, and AIPTW: 1.8% bias), but ELTMLE produces an estimate that is unbiased (ie, ELTMLE: 0% bias to 3 decimal places) relative to the true ATE. The relative bias from only one simulated sample for the regression adjustment and IPTW estimator is large because they rely on the positivity assumption, which, in this simulation, is violated because there was a low number of individuals with a higher comorbidity value. Without correcting for this imbalance in the data, the methods that rely on this assumption will be vulnerable to bias.

## 8 | CONCLUSION

Overall, methods introduced here rely on the estimation of the g-formula (nonparametrically or parametrically), which is a generalization of standardization, the inverse probability of treatment weighting (IPTW), or their combination (ie, double-robust methods).^3^ However, there are other estimators based on matching strategies that we did not cover here.^19^ Readers can find a more detailed overview of the propensity score and matching methods in a recently published article.^48^

Table 2 shows the results of the ATE for all of the different causal inference estimators we introduced in the tutorial. Overall, all of the methods showed a consistent result for the ATE (Table 2). The RHC data (demonstrated in this article) is used to teach causal inference methods because of its extremely well-balanced distribution of confounders across levels of the treatment (RHC). However, in most observational studies, data are not usually well-balanced and there are potentially near violations of the positivity assumption that must always be checked.

We introduced different estimators in regards to their chronological development: the methods were developed to answer the limitations of the previous approach. For example, parametric estimators were developed to address the curse of dimensionality. Then, issues related to extrapolation for the G-computation, and the instability of the estimations due to large weights for the IPTW estimators, encouraged the development of double-robust methods. AIPTW was a strong candidate to answer this issue by incorporating semi-parametric theory and methods to causal inference. However, it was known that it did not solve the estimation equation (ie, equal to zero) due to the fact that it is not a substitution estimator or plug-in estimator (see Glossary). Thus, to overcome this limitation of the AIPTW estimator, data-adaptive estimation using machine learning algorithms and ensemble learning to estimate the nuisance parameters from the regression and propensity score models, were combined to solve the estimation equation.^4^ Evidence shows that the double-robust estimators (particularly TMLE) obtain less biased estimates of the true causal effect in comparison to naive estimators such as multivariate regression.^4^

Evidence shows that when comparing the underlying properties of each method based on Monte-Carlo experiments, only TMLE provides the numerous properties of estimating the probability distribution that enable it to out-perform the others. The properties of the estimator are: loss-based, well-defined, unbiased, efficient and can be used as a substitution estimator. Maximum likelihood estimation (MLE) based methods (stratification, propensity score and parametric regression) and other estimating equations (IPTW and AIPTW) do not have all of the properties of TMLE and evidence shows that they underperform in comparison to TMLE in selected samples. For more detailed comparisons between the different methods, the interested reader is referred to Chapter 6 of van der Laan and Rose’s TMLE textbook.^4^ It is important to highlight that in contrast to the AIPTW estimator, TMLE respects the global constraints of the statistical model (ie, $P_{0}(0<Y<1)=1$) and solves the estimation equations being equal to zero.^4^

However, even if TMLE is less prone to errors due to misspecification than alternative methods (eg, inverse probability weighting) there is some question regarding the validity of the robustness of inference produced by TMLE in nonparametric settings.^49^ This is an area of ongoing work (ie, double/debiased machine learning, cross-validated TMLE and cross-fit estimators).^44,50,51^ Furthermore, TMLE and the *SuperLearner* were originally developed in R.^35,46^ Outside R, there is a Python library implementing TMLE and the *SuperLearner* named *zEpid*,^52^ and a SAS library implementing the *SuperLearner*.^53^ Also, there is a user written program for Stata (*eltmle*).^45^ However, *eltmle* is not completely native to Stata but rather calls the *SuperLearner* R package to calculate the predictions of the treatment and outcome models. More work is required to continue implementing and improving the TMLE framework in other statistical software.^35^

Causal inference is a growing field in rapid developments. Modern causal inference methods allow machine learning to be used when strong assumptions for parametric models are not reasonable. Overall, due to the difficulty of correctly specifying parametric models in high-dimensional data, we advocate for the use of double-robust estimators with ensemble learning. Using these approaches may require larger sample sizes to avoid finite-sample bias.^16,54^ However, recent developments support the use of cross-fit double-robust estimators for data adaptive estimation.^44,50^ Tutorials introducing the use and derivation of the functional delta method and influence curve for applied researchers are needed. The tutorial presented here may help applied researchers to gain a better understanding of the computational implementation for different causal inference estimators.

## Supplementary Material

Supplement 1

## Figures and Tables

**TABLE 1:** Estimates of ATE from the different computational methods

Method	ATE	95% CI	ATE	Bootstrap 95% CI
*One confounder*				
Regression	7.35	4.84 to 9.86	7.35	4.78 to 9.92
NPG-1C	n/a	n/a	7.37	4.72 to 9.89
NPG-FS	7.37	4.83 to 9.91	7.37	4.68 to 9.84
PG-1C	7.37	4.83 to 9.91	7.37	4.68 to 9.84
*Multiple confounders*				
Regression	8.26	5.77 to 10.75	8.26	5.69 to 10.83
PG-FS	8.36	5.83 to 10.88	8.36	5.68 to 10.84
IPW-PS	8.33	5.81 to 10.85	8.33	5.65 to 10.81
MSM	8.33	5.77 to 10.89	8.33	5.74 to 10.62
IPW-RA	8.35	5.82 to 10.87	8.35	5.74 to 10.63
AIPW	8.35	5.82 to 10.87	8.39	5.78 to 10.66
TMLE	8.45	5.92 to 10.97	n/a	n/a
ELTMLE	8.35	5.82 to 10.87	n/a	n/a

*Note*: n/a, 95% CI were not computed for the NPG-1C because the normal approximation was not appropriate. Bootstrap 95% CIs for the TMLE and ELTMLE estimators are not theoretically supported.

Abbreviations: 1C, one confounder; AIPW, augmented inverse probability weighting; ELTMLE, ensemble learning targeted maximum likelihood estimation using Stata *eltmle* package; FS, fully saturated; IPW, inverse probability weighting; MSM, marginal structural model; NPG, nonparametric g-formula; PG, parametric g-formula; PS, propensity score; RA, regression adjustment; TMLE, targeted maximum likelihood estimation by hand.

**TABLE 2:** Distribution of the treatment before and after applying weights

	Standardized differences		Variance ratio	
Confounder	Raw	Weighted	Raw	Weighted
Sex	0.093	0.000	0.977	1.000
Age	−0.061	−0.004	0.817	0.791
Education	0.091	−0.002	1.015	1.027
Race: Black	−0.031	0.002	0.944	1.003
Race: Other	0.020	0.001	1.078	1.004
Cancer: Metastatic	−0.069	−0.000	0.780	1.000
Cancer: Localized	−0.072	0.000	0.879	0.999

*Note*: Reference groups (race: white, cancer: none).

## GLOSSARY

The glossary is adapted from the book *Targeted learning: causal inference for observational and experimental data* and a recent publication introducing the TMLE framework.^4,55^

Data-generating process: The mechanism that generated the observed data, with the corresponding data-generating probability distribution which produces the observed samples that were collected
Estimand: A quantity we are interested in estimating from our data
Estimator: A function of the sample of observations (ie, a function of the random variables) that generates estimates. The estimators are represented by algebraic equations that explicitly describe a function of the realized observations
Estimate: The realized value of an estimator, or a function of the realized observations. It is the value of the quantity defined by the estimand
Counterfactual: A contrary-to-fact value said to arise from hypothetically imposing an intervention on a system represented by a structural causal model. For example, the potential outcome Y(a) is a counterfactual that arises from a hypothetical intervention that sets the treatment A to level a
Statistical model: A set (family) of probability distributions that could describe the data-generating process (DGP). Note that, outside simulation exercises, the true DGP is unknown
Saturated model: A saturated model fits the data perfectly and it includes the main terms plus the higher order interactions between the factors included in the model. Usually, the number of parameters is equal to the number of the possible combinations between the levels of the distinct covariates included in the model
Model misspecification: A scenario in which the statistical model, which is postulated to contain the distribution describing the DGP, fails to actually contain the corresponding true data-generating distribution
Parametric statistical model: A family of probability distributions indexed by a finite set of model parameters. For example, a linear model traditionally assumes the outcome is a linear function of covariates plus a normally distributed error term with constant variance. Its parameters are the coefficients on the covariates and the variance of the error term
Nonparametric statistical model: A family of probability distributions that cannot be indexed by a finite set of parameters. That is, the set of parameters indexing this family of distributions is infinite-dimensional. Most often, when making minimal assumptions, the DGP cannot be defined by a finite set of parameters, making the set of parameters infinite-dimensional. For example, if all we know about the DGP is that we have access to *n* independent and identically distributed (i.i.d.) samples, then the statistical model for the DGP is a nonparametric statistical model
Target estimand or target parameter: A function of the true (unknown) DGP that one is interested in estimating, and represents the mathematical formulation of the motivating question of interest
Maximum likelihood estimation: The most common method for estimating parameters in a finite-dimensional model (ie, parametric statistical model). As the name implies, such estimates are generated by finding a set of parameter estimates that maximize the likelihood function of the observed data
Score equation: The gradient (ie, multi-variable generalization of the derivative) of the log-likelihood function of the data with respect to the parameter(s). This equation provides information on the degree of change resulting from very small perturbations of the parameter values
Regular estimator: A class of estimators that converge in distribution to some limit distribution even if one samples from a slightly perturbed data distribution. Such estimators, if also asymptotically linear, accommodate inference by way of their asymptotic convergence to a Normal distribution
Plug-in (substitution) estimator: An estimator that generates an estimate of the true parameter value by “plugging in” estimates of relevant parts of the data-generating distribution into the parameter mapping. This method is commonly referred to as the plug-in principle. For example, “plugging in” targeted Super Learner fit of the conditional mean under A=1 and A=0 generates an estimate of the average treatment effect
