# Supplementary material for: Introduction to computational causal inference using reproducible Stata, R, and Python code: A tutorial
Source: Stat Med. Author manuscript; Available in PMC 2025 Feb 5. (PMC11795351; doi:10.1002/sim.9234)
Supplement: Supplement 1 [file NIHMS1890988-supplement-Supplement_1.pdf]

1 | SUPPLEMENTARY WEB MATERIALS

1.1 | Appendix 1: Potential outcomes framework, causal assumptions, and g-formula

To illustrate the framework we use an empirical example based on intensive care medicine.[1] The study, set in intensive care units of five United States teaching hospitals between 1989 and 1994, evaluated the effectiveness of right heart catheterisation (RHC) on short-term mortality (30 days) of 5,735 critically ill adult patients (2,184 received a RHC and 3,551 did not received it) receiving care for 1 of 9 prespecified disease categories. In our illustration, the outcome is short-term mortality defined as 30 days after ICU admission, and RHC was the main intervention henceforth the treatment, and we define ($\boldsymbol{W}$) to include the set of confounders. Let Y denote the vital status of the patient in an intensive care unit (ICU) at 30 days after admission. Let A denote the treatment of whether or not the patient received RHC during their stay at the ICU, and let C denote a binary confounder.

For a binary treatment, each patient in the study has two potential outcomes (i.e., Y(a)), where Y(1) denotes the potential outcome if they received RHC, and Y(0) denotes the potential outcome if they did not receive RHC.[2] However, only one of the potential outcomes can be observed since a patient can only ever receive one of the treatments and only one of the outcomes (they cannot both live and die after 30 days). As an example from Table 1, imagine that Matthew has two potential outcomes: firstly, $Y(0) = 1$ says that if Matthew were not to receive RHC then he would die within 30 days, and secondly, $Y(1) = 0$ says that if Matthew were to receive RHC then he would not die within 30 days. Likewise, for the rest of the individuals their potential outcomes are presented and the ATE can be estimated as the contrast between the *potential outcomes* under different treatment levels (i.e., the difference between E[Y(1)] - E[(Y(0)]).[3]

**Patient**	**Y**	**A**	**C**	Y(0)	Y(1)
Matthew	1	0	0	1	0
Camille	1	1	1	1	1
Aurelien	1	1	1	0	1
Paul	0	1	0	0	0
Mohammad	1	0	1	1	1
Steve	0	1	1	0	0
Miguel	1	0	0	1	1
Bernard	0	1	1	0	0
Clemence	1	1	0	1	1

TABLE 1 Potential outcomes framework: C = Binary confounder, A = Binary treatment, Y = Binary outcome, Y(0) = Potential outcome when untreated, Y(1) = Potential outcome when treated

However, we must make certain assumptions to identify potential outcomes from the observed data and then estimate the ATE.[4] Given that the potential outcomes are not necessarily directly observed from the data, to identify the ATE from observable data (i.e., from Table 1) the following three assumptions are made:

1. Counterfactual consistency

Counterfactual consistency holds if the observed outcome for all treated individuals equals their outcome if they had been treated, and likewise for untreated individuals. For example, in Table 1, Matthew's observed outcome equals his potential outcome if he had not been treated ($Y = Y(0) = 1$). This means that the definition of the treatment, and outcome, is consistent for Matthew (the same applies for all the other patients). Analytically, consistency is represented by:

$$Y = AY(1) + (1 - A)Y(0)$$

We further assume observations are independent (e.g., no interference) and there is no measurement error.

2. Conditional exchangeability

In randomised studies, conditional and marginal exchangeability holds because the treated individuals, had they not been treated, would have had the same average potential outcomes as the untreated, and vice versa. This cannot be guaranteed in observational studies but it can be assumed to hold if the unmeasured risk factors of the outcome are equally distributed between the treated and the untreated groups conditional on the measured confounders. Thus, using the language of the potential outcomes, the conditional exchangeability assumption (a.k.a conditional independence, unconfoundedness or ignorability) is given:

$$\mathrm{Y(a)} \amalg \mathrm{A} \mid \mathbf{C} \ \forall \ \mathrm{a} \in \{0, 1\}$$

Hence, the conditional mean independence is given

$$\mathrm{E[Y_a \mid A = 1, C = c] = E[Y_a \mid A = 0, C = c] = E[Y_a \mid C = c]} \ \forall \mathrm{a} \in 0, 1$$

3. Positivity

Positivity holds if the conditional probability of being treated (and similarly for being untreated) is greater than zero. Therefore, if $\mathrm{P(C = c) > 0}$ then $\mathrm{P(A = a \mid \mathbf{C} = c) > 0} \ \forall \ \mathrm{C} \in \mathbf{c}, \mathrm{a} \in \{0, 1\}$. When this assumption is violated, it is typically because the target population is poorly defined (trying to estimate the effect of a treatment on people who would never receive it anyway).

With these assumptions, the observed data can then be used to estimate the average treatment effect as follows:

By the law of total probability

$$\mathrm{P[Y(a) = 1] = \sum_c P[Y(a) = 1 \mid C = c]\, P(C = c)}$$

By conditional exchangeability the right hand side is

$$\mathrm{\sum_c P[Y(a) = 1 \mid A = a, C = c]\, P(C = c)}$$

This is possible since we are assuming that, within levels of *C*, the predictors of the outcome are equally distributed between treated (e.g., RHC) and non-treated (e.g., non-RHC) groups: that is we have achieved what would happen if patients were randomised to each treatment within stratum of C. If we assume consistency the right hand side is

$$\mathrm{\sum_c P[Y = 1 \mid A = a, C = c]\, P(C = c)}$$

The ATE is defined as

$$\mathrm{P(Y(1) = 1) - P(Y(0) = 1)}$$

and, under the preceding assumptions, can be estimated by

$$\mathrm{\sum_c P[Y = 1 \mid A = 1, C = c]\, Pr(C = c) - \sum_c P[Y = 1 \mid A = 0, C = c]\, P(C = c).} \quad (1)$$

We have transitioned from the (unobserved) potential outcomes to a setting where we can estimate our causal estimand, from the distribution of the observed data, using equation 1, namely the g-formula.[5]

1.2 | Appendix 2: Equivalence between IPTW and G-computation

By repeated use of the law of total expectation, the IPTW and the G-computation regression adjustment estimators for the ATE are equivalent as given by

$$\underbrace{E\left(\frac{I(a=1)}{P(A=1\mid \mathbf{W})}Y\right)}_{\text{IPTW}} =$$

By definition of expectations...

$$= \sum_{w,a,y} \frac{I(a=1)}{P(A=1|W=w)}\, y\, P(Y=y, A=a, W=w)$$

By the law of total probability...

$$= \sum_{w,a,y} \frac{I(a=1)}{P(A=1\mid W=w)}\, y\, P(Y=y\mid A=a, W=w)\, P(A=a\mid W=w)\, P(W=w)$$

Cancellation by evaluating at A=1...

$$= \sum_{w,y} y\, P(Y=y\mid A=1, W=w)\, P(w=w)$$

By definition of expectations...

$$= \sum_{w} E(Y\mid A=1, W=w)\, P(W=w)$$

Finally, again by definition of expectations...

$$= \underbrace{E[E(Y\mid A=1, W)]}_{\text{G–computation}}$$

References

1. Connors Alfred F., Speroff Theodore, Dawson Neal V., et al. The effectiveness of right heart catheterization in the initial care of critically ill patients. *Journal of the American Medical Association.* 1996;276(11):889–897.

2. Rubin Donald B. Estimating causal effects of treatments in randomized and nonrandomized studies.. *Journal of educational Psychology.* 1974;66(5):688.

3. Rubin Donald B. Causal inference using potential outcomes. *Journal of the American Statistical Association.* 2005;100(469):322–331.

4. Robins James. A new approach to causal inference in mortality studies with a sustained exposure period—application to control of the healthy worker survivor effect. *Mathematical Modelling.* 1986;7(9):1393–1512.

5. Robins James M.. Association, Causation, and Marginal Structural Models. *Synthese.* 1999;121(1/2):151–179.

□
